# Performance of SAPS II and SAPS 3 in Intermediate Care

**DOI:** 10.1371/journal.pone.0077229

**Published:** 2013-10-09

**Authors:** Juan F. Lucena, Félix Alegre, Diego Martinez-Urbistondo, Manuel F. Landecho, Ana Huerta, Alberto García-Mouriz, Nicolás García, Jorge Quiroga

**Affiliations:** 1 Clínica Universidad de Navarra, Department of Internal Medicine, Division of Intermediate Care and Hospitalists Unit, Navarra, Pamplona, Spain; 2 Clínica Universidad de Navarra, Information Technology, Pamplona, Spain; Rutgers University, United States of America

## Abstract

**Objective:**

The efficacy and reliability of prognostic scores has been described extensively for intensive care, but their role for predicting mortality in intermediate care patients is uncertain. To provide more information in this field, we have analyzed the performance of the Simplified Acute Physiology Score (SAPS) II and SAPS 3 in a single center intermediate care unit (ImCU).

**Materials and Methods:**

Cohort study with prospectively collected data from all patients admitted to a single center ImCU in Pamplona, Spain, from April 2006 to April 2012. The SAPS II and SAPS 3 scores with respective predicted mortality rates were calculated according to standard coefficients. Discrimination was evaluated by calculating the area under receiver operating characteristic curve (AUROC) and calibration with the Hosmer-Lemeshow goodness of fit test. Standardized mortality ratios (SMR) with 95% confidence interval (95% CI) were calculated for each model.

**Results:**

The study included 607 patients. The observed in-hospital mortality was 20.1% resulting in a SMR of 0.87 (95% CI 0.73-1.04) for SAPS II and 0.56 (95% CI 0.47-0.67) for SAPS 3. Both scores showed acceptable discrimination, with an AUROC of 0.76 (95% CI 0.71-0.80) for SAPS II and 0.75 (95% CI 0.71- 0.80) for SAPS 3. Calibration curves showed similar performance based on Hosmer-Lemeshow goodness of fit C-test: (X^2^=12.9, p=0.113) for SAPS II and (X^2^=4.07, p=0.851) for SAPS 3.

**Conclusions:**

Although both scores overpredicted mortality, SAPS II showed better discrimination for patients admitted to ImCU in terms of SMR.

## Introduction

Worldwide health care institutions try to give care based on best-practice models with cost-effectiveness. In this scenario, intermediate care units (ImCU) provide a rational and proportional treatment between the intensive care and the general ward.

Previous studies suggested that around 35% of intensive care unit (ICU) admissions were for low risk patients that were admitted mainly for monitoring purposes [1,2], while, in contrast, some patients were treated on general wards when they should receive more intensive care and monitoring [3]. As a result, the development of intermediate care showed encouraging results in terms of cost-containment and ICU utilization, triage flexibility for acute patients and mortality rates for hospital wards [3-10].

Nevertheless, the characteristics of the ImCUs and the type and amount of services provided, varies depending on resource availability, institutional infrastructure and the parent health care system. Accordingly, the case mix population of intermediate care may show great heterogeneity.

Characterization of these patients relies on the assessment of their illness severity, using severity scores. Although the performance of severity scores has been widely described in ICU patients, the information in the setting of ImCU is very limited [11-13]. The purpose of this study was to analyze the performance of both, the Simplified Acute Physiology Score (SAPS) II and SAPS 3 in a single center ImCU.

## Materials and Methods

This study was performed at the Clínica Universidad de Navarra, an academic medical center of 300 beds, in Pamplona, Spain. Its ImCU is a 9-bed multi-purpose unit adjacent to, but independent from, the mixed ICU. Each bed is equipped with continuous telemetry, pulse oxymetry, noninvasive arterial blood pressure, central venous pressure monitoring, and noninvasive pressure support ventilation. The signals are relayed to a central monitoring station and the nurse-to-patient ratio is 1:3. The ImCU infrastructure (beds, technical resources and nursing staff) is shared by the Stroke Unit and the Coronary Care Unit. These units also have independent admission criteria and medical staff, and the patients from these units were not taken into account for the present study. The ImCU rounding team involves a nurse, the hospital pharmacist, the ImCU resident, the specialist or surgeon and the attending hospitalist. The hospitalist was responsible for admission and discharge of all ImCU patients. Admission and discharge criteria for the ImCU were set according to previous guidelines defined by The American College of Critical Care Medicine [14], and also served as inclusion criteria for the present study. Exclusion criteria included: age less than 18 years old, severe respiratory failure at imminent risk of requiring intubation, status epilepticus, and catastrophic brain illness. ImCU readmissions and patients admitted for drug administration and desensitization, were excluded from data analysis. Patients came from medical and surgical wards, ICU, the operating room, and the emergency room.

From April 2006 to April 2012, every consecutive patient admitted to the ImCU was evaluated. Demographics, past medical history, reasons for admission, physiological parameters at the time of admission and during the first 24 hours of ImCU stay, laboratory variables and survival to hospital discharge were prospectively recorded by the authors. Patients with missing variables were also excluded from data analysis.

The SAPS II and SAPS 3 scores with their respective predicted mortality rates were calculated according to standard coefficients [15,16]. We did not use the SAPS 3 customized equation for Southern Europe, since previous studies showed similar results using the general equation [17]. Data considered for the calculation of SAPS 3 and SAPS II scores were recorded within 1 hour and during the first 24 hours of ImCU admission, respectively. In-hospital mortality was the end-point of the study.

### Ethics Statement

The study protocol was approved by the Institutional Review Board (IRB) at the Clínica Universidad de Navarra (ref. 129/2010). The IRB waived the need for informed consent, because it is an observational non-interventional cohort study with prospectively collected data and retrospective analysis, and also because it did not interfere with decisions related to patient´s care. The study has been performed in accordance with the ethical standards laid down in the 1964 Declaration of Helsinki and its later amendments.

### Statistical Analysis

Data were entered into a computer data base by the authors. Statistical analysis was performed using SPSS for Windows, version 15.0 (SPSS Inc, Chicago, IL). Continuous variables were reported as mean ± standard deviation or median (25%-75% interquartile range). For nonparametric measure of statistical dependence of quantitative variables, we used Spearman´s correlation coefficient. Categorical variables were reported as absolute numbers (frequency percentages) and analyzed by chi-square test. Validation of the prognostic scores was assessed by standard tests to measure discrimination and calibration. Discrimination was evaluated by calculating the area under receiver operating characteristic curve (AUROC) [18] and calibration with the Hosmer-Lemeshow goodness-of-fit C test [19]. A high p value (> 0.05) would indicate a good fit for the model. Calibration curves were constructed by deciles of predicted mortality (x-axis) against observed mortality (y-axis). Standardized mortality ratios (SMR) (ratio of observed to expected deaths) with 95% confidence interval (95% CI) were calculated for each model. 

## Results

During the study period, 938 patients were admitted in the ImCU. Of these, 331 were excluded: 66 low risk patients (drug administration and desensitization), 170 readmissions, and 95 patients for missing variables. Six hundred and seven (607) patients were included for data analysis.

Patient characteristics, location prior to admission, surgical status, coomorbidities and discharge location are described in Table 1.

**Table 1:** Patient Characteristics and Mortality (n 607).

**Age (years)**	66±14
**Gender**	
**Male**	373 (61.4%)
**Female**	234 (38.6%)
**Location prior to admission:**	
**General Ward**	315 (51.9%)
**Emergency Room**	151 (24.9%)
**ICU**	85 (14.0%)
**Operating room**	38 (6.3%)
**Other hospital**	18 (2.9%)
**Coomorbidities in SAPS 3**	
**Immunosupression**	235 (38.7%)
**Metastatic cancer**	140 (23.1%)
**Haematological cancer**	33 (5.4%)
**Cirrhosis**	42 (6.9%)
**Chronic heart failure. NYHA IV**	31 (5.1%)
**Surgical status**	
**Planned**	47 (7.7%)
**Emergency**	31 (5.1%)
**Discharge Location**	
**Death**	36 (5.9%)
**General Ward**	473 (78.0%)
**ICU**	75 (12.3%)
**Home**	5 (0.8%)
**Other Hospital**	18 (3.0%)
**Death Location**	
**ImCU**	36/607 (5.9%)
**ICU**	38/75 (50.7%)
**General Ward**	48/473 (10.1%)

**ICU**: Intensive Care Unit. **SAPS 3**: Simplified Acute Physiology Score 3.

**ImCU**: Intermediate Care Unit. **NYHA** IV: New York Heart Association Class IV.

The mean age was 66 years with 61% male. The patients were admitted from general ward (52%), emergency room (25%), ICU (14%), operating room (6%) and a small number of patients came from other hospital wards (3%). Reasons for admission were essentially medical (87%), with respiratory failure (33%) and sepsis (21%) as the most important causes (Table 2). The average length of stay was 5±6 days.

**Table 2:** Reasons for ImCU admission and SMR based on SAPS II and SAPS3.

**Condition**	**Patients**	**SAPS II**	**SMR (95%CI)**	**SAPS3**	**SMR (95%CI)**
**Respiratory failure**	199	36.1 +/- 9.3	1.09 (0.81-1.44)	62.6 +/- 12.4	0.55 (0.4-0.73)
**Sepsis**	129	44.8 +/- 13.8	0.71 (0.49-1.00)	67.6 +/- 12.8	0.52 (0.36-0.73)
**Cardiovascular**	82	35.3 +/- 10.5	1.05 (0.64-1.64)	54.7 +/- 14.0	0.78 (0.48-1.21)
**Perioperative**	79*	29.1 +/- 9.8	0.39 (0.10-0.89)	43.0 +/- 14.5	0.34 (0.09-0.74)
**Complex monitoring**	51	33.4 +/-11.8	0.82 (0.34-1.58)	51.4 +/- 13.5	0.64 (0.28-1.21)
**GI complications**	41	31.6 +/- 7.9	0.66 (0.17-1.48)	55.2 +/- 12.5	0.35 (0.09-0.81)
**Neurologic**	16	40.1 +/- 8.9	1.59 (0.50-2.67)	61.0 +/-13.8	1.17 (0.36-1.96)
**Liver Failure**	10	41.0 +/- 14.9	1.38 (0.35-2.96)	60.1 +/-20.2	0.96 (0.26-2.20)

**ImCU**: Intermediate Care Unit. **SMR**: Standardized mortality ratios.

**SAPS**: Simplified Acute Physiology Score. **CI**: Confidence interval.

* The number of perioperative patients is not the same of the surgical status subgroup described in Table 1 (n78), because some perioperative patients were admitted for medical reasons and the surgery was not the main cause.

**GI**: Gastrointestinal complications.

**Figure 1:**
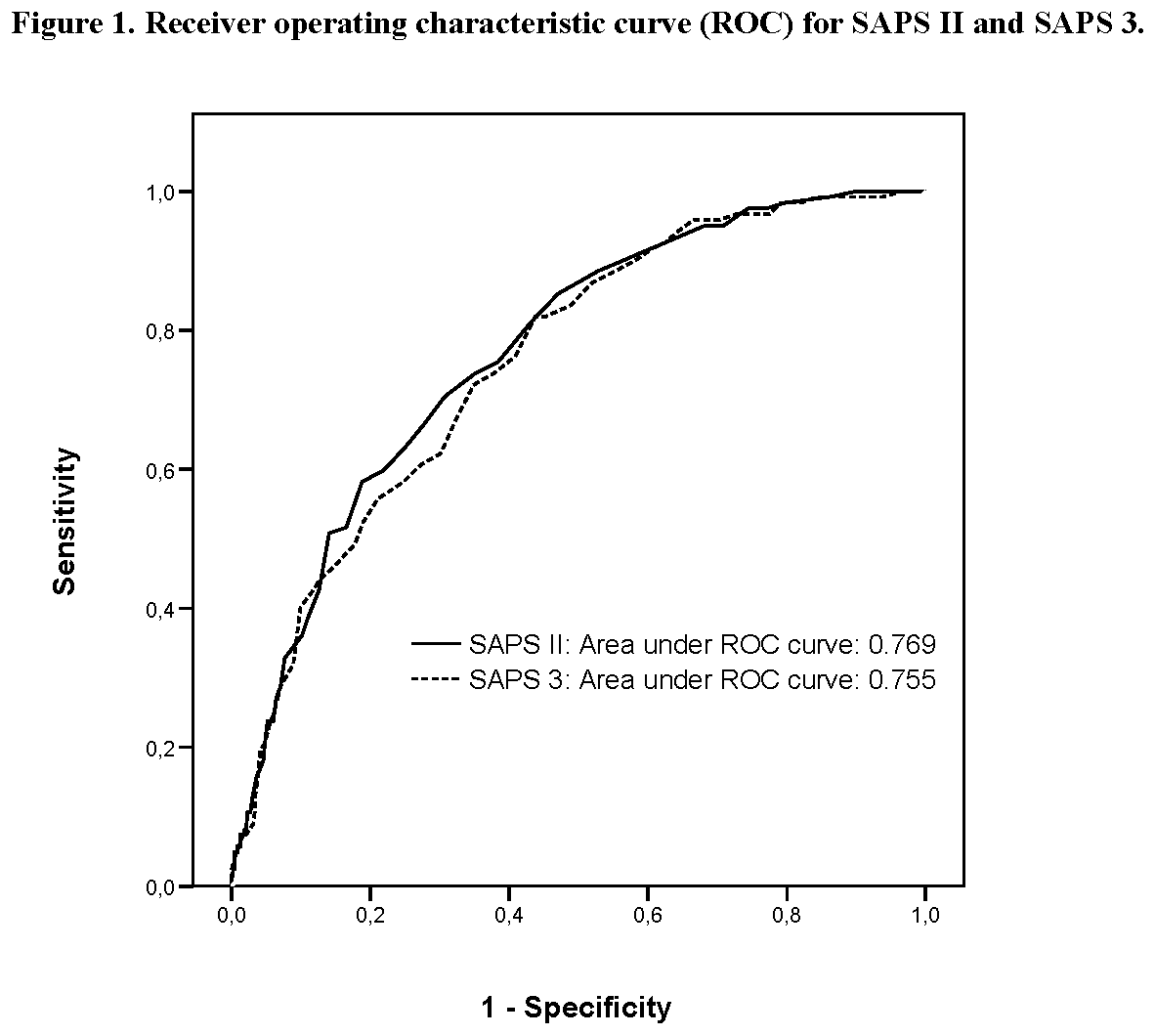
Receiver operating characteristic curve (ROC) for SAPS II and SAPS 3.

**Figure 2:**
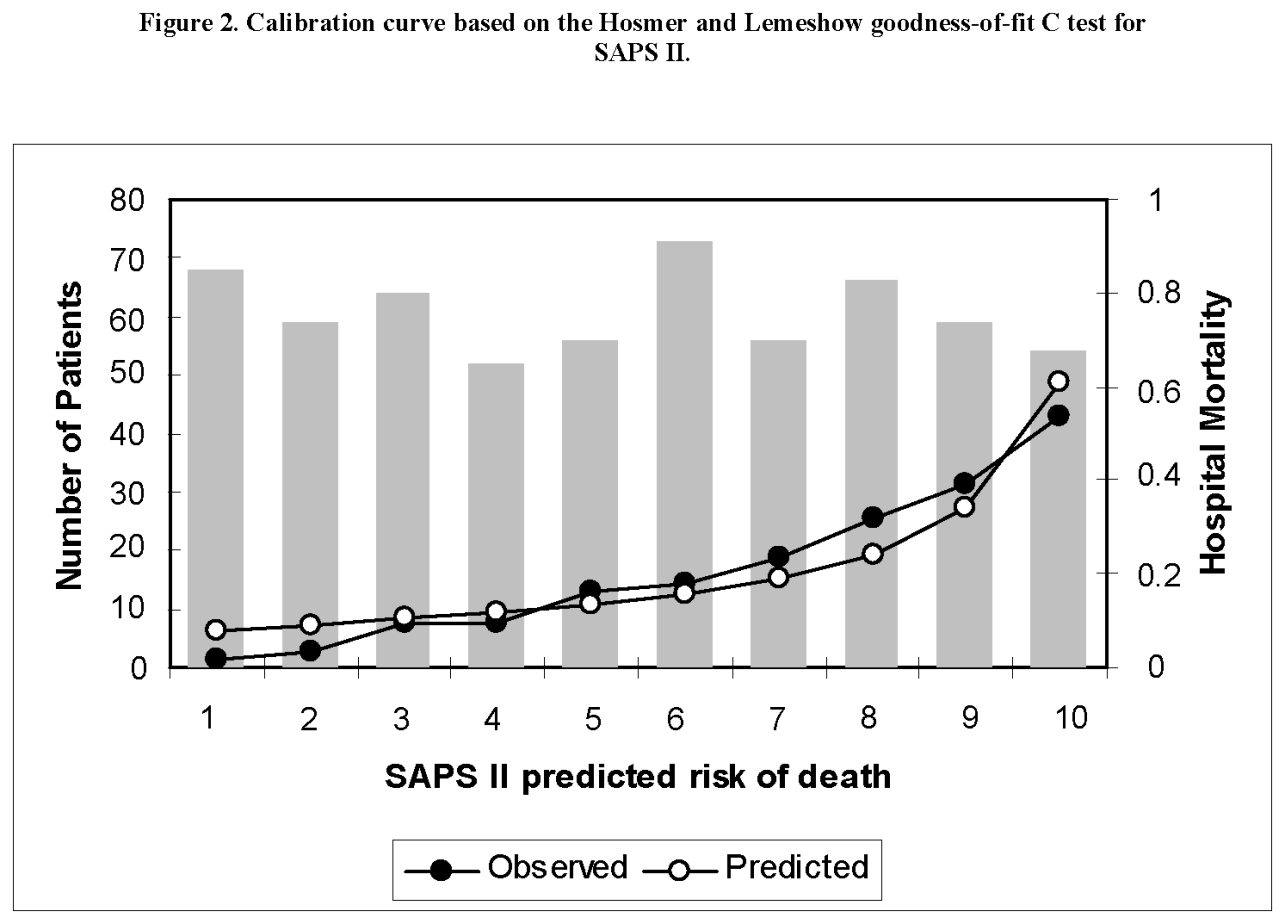
Calibration curve based on the Hosmer and Lemeshow goodness-on-fit C test for SAPS II.

**Figure 3:**
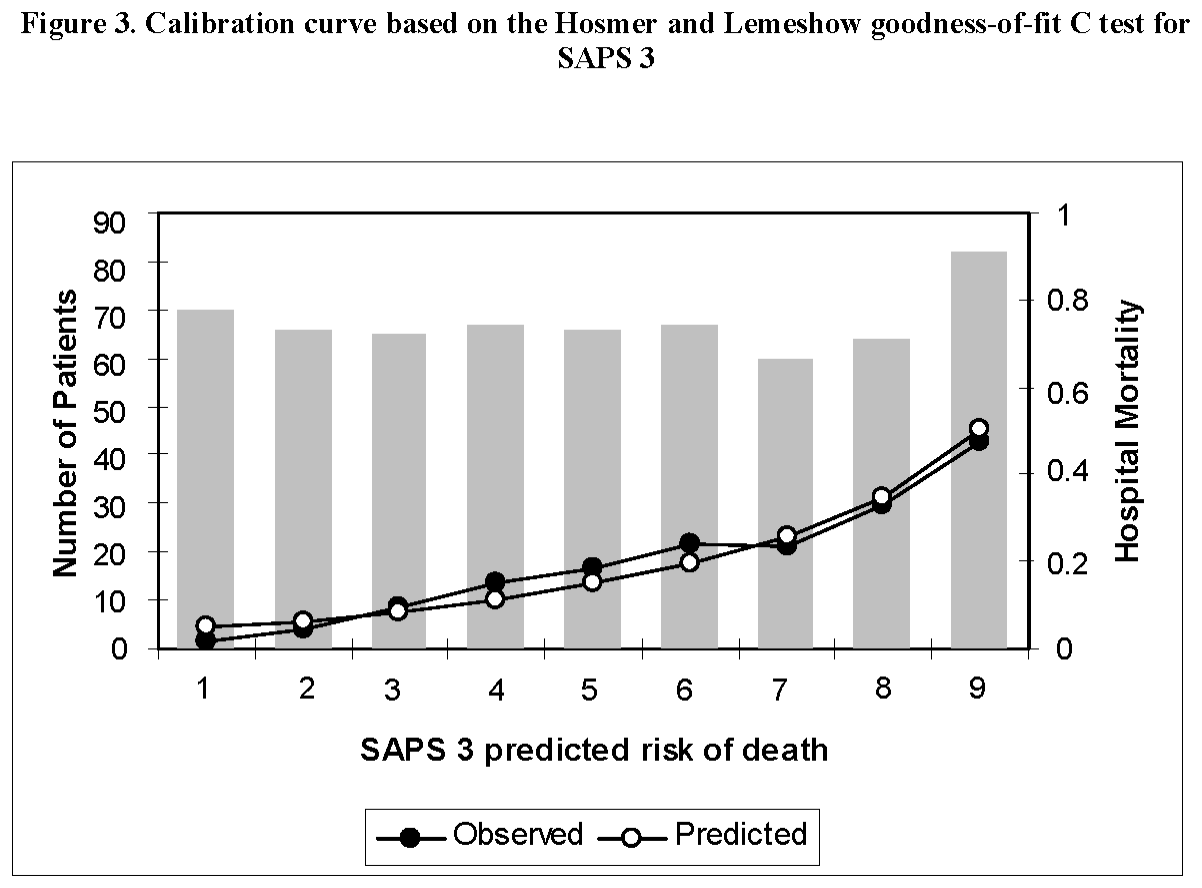
Calibration curve based on the Hosmer and Lemeshow goodness-on-fit C test for SAPS 3.

The mean SAPS II and SAPS 3 of the cohort were 36.6±11.9 and 58.4±15.4 respectively, and the expected mortality derived from these scores were 22.9 % and 35.6%. The observed in-hospital mortality was 20.1% (122/607) resulting in a SMR of 0.87 (95% CI 0.73-1.04) for SAPS II and 0.56 (95% CI 0.47-0.67) for SAPS 3. Performances of the models are presented in Table 3. Both scores showed acceptable discrimination, with an AUROC of 0.76 (95% CI 0.71-0.80) for SAPS II and 0.75 (95% CI 0.71-0.80) for SAPS3 (Figure 1). Although both scores overpredicted mortality, SAPS II showed better discrimination with results closer to the observed mortality based on SMR. Nonetheless, the discrimination performance assessed by AUROC did not show any meaningful difference. Calibration curves are presented in Figures 2 and 3. Both scores showed similar calibration performance based on Hosmer-Lemeshow goodness of fit test: (X^2^=12.9, p=0.113) for SAPS 2 and (X^2^=4.07, p=0.851) for SAPS 3. Nonetheless, there is a non-significant trend for better calibration performance for SAPS 3. We also established a significant correlation between SAPS II and SAPS 3 in predicting mortality (Rho 0.66 p < 0.01). 

**Table 3:** Performance of SAPS II and SAPS 3 scores in ImCU.

	**Score**	**Predicted mortality**	**SMR**	**Goodness-of-fit C-test**		**AUROC**
		**(SD)**	**(95%CI)**	**X^2^**	**p value**	**(95%CI)**
SAPS II	36.6±11.9	22.9±18.5	0.87 (0.73-1.04)	12.9	0.113	0.76 (0.71-0.80)
SAPS 3	58.4±15.4	35.6±23.9	0.56 (0.47-0.67)	4.07	0.851	0.75 (0.71-0.80)

**SAPS**: Simplified Acute Physiology Score. **ImCU**: Intermediate Care Unit.

**SD**: standard deviation. **SMR**: Standardized mortality ratios. **CI**: Confidence interval. **AUROC**: Area under receiver operating characteristic curve.

## Discussion

Although intermediate care is worldwide recognized, there are few studies in the last decades demonstrating the efficacy of these areas [3-12]. Moreover, there are only two previous descriptions of SAPS II in this setting [11,12]. Auriant et al. described in 1998, the performance of SAPS II in a cohort of 433 patients, showing good discriminant power (AUROC 0.85 ± 0.04) and calibration (C=2.4, p> 0.5). The SMR of the cohort was 0.93, with an observed mortality of 8.1% (11). Our group recently described similar results, with considerably higher mortality rates in a cohort of 456 patients. The observed mortality of the cohort was 20.6% with an expected mortality derived from SAPS II calculation of 23.2% (SMR 0.89) (AUROC 0.75, p< 0.001) [12].

To the best of our knowledge, this is the first external validation study of the SAPS 3 score in ImCU population. In our cohort, this relatively new score was not superior to its previous version. Furthermore, the performance of SAPS II showed better discrimination with results closer to the observed mortality.

It is noteworthy the high risk of the patients in our study, based on elevated SAPS II (36.6±11.9) and SAPS 3 (58.4±15.4) scores, with considerable expected mortality rates 22.9% and 35.6% respectively. The contribution of the subgroup of oncologic patients (187/607), with advanced disease (75%), and elevated SAPS II (42.1±12.2) and SAPS 3 (67.2±14.1) scores, could explain in part these findings. The admission of these patients was required because their acute disease needed close monitoring and was not due to ICU bed restriction. However, in some cases, they were not candidates for ICU admission due to the underlying disease, chronic coomorbidities or advanced age.

Observed to expected mortality ratios (SMRs) have become standard tools for assessing the impact of ICU-related outcomes, including unit organization and management [20]. Therefore, it is very important to evaluate the performance of prognostic models that are used to generate these data and finally detecting potential biasses. Poor calibration and mortality underestimation could prevent relevant comparisons of data extracted from SMRs. In addition, external validation studies are required before applying new scores to other case-mixes. Soares and Salluh [21], found excellent discrimination for SAPS II and SAPS 3 in a cohort of 952 oncologic patients admitted to the ICU. The calibration of SAPS 3 and its customized equation for Central and South American countries in this cohort was appropriate, but the calibration of SAPS II was poor. These results were in agreement with other reports on the performance of SAPS II in ICU patients from European countries, in which the model failed to adjust adequately for differences in the case-mix profiles [22-24]. 

In the present study, we found similar calibration performance of the models, with a non-significant trend for better performance for SAPS 3, based on the Hosmer Lemeshow goodness of fit C-test. However, it is surprising the significant mortality overprediction based on the original SAPS 3 (SMR 0.56, 95% CI 0.47-0.67), and compared to SAPS II (SMR 0.87, 95% CI 0.73-1.04, closer to unit).

Changes in case mix in critical care, has been one of the reasons related to the poor calibration of SAPS II over time and one of the arguments for the development of new generation scores. It is possible that the oldest score avoids the potential overfitting bias of SAPS 3, when the validation case mix cohort is substantially different from the original study. Additionally, recent external validation studies in ICU population, showed similar results to ours, with both scores overpredicting mortality, but SAPS 3 more than SAPS II [25,26]. 

These conflicting results emphasize the need to find more reliable and accurate scores for intermediate care patients, based on larger, prospective and well designed studies.

The present study provides an external validation of the SAPS 3 score in intermediate care. However, several limitations must be addressed. The case-mix of our cohort may differ from that in other ImCUs, limiting the extrapolation of the results to other populations. Case-mix differences between the development cohort of SAPS 3 and our population could deteriorate calibration and the ratios of observed to expected mortality [27]. The small sample size derived from a single center study, and even more restricted samples of various subgroups of the population, could interfere with the evaluation of the uniformity of fit among different expected mortality subgroups. Moreover, the traditional calibration methods, such as Hosmer-Lemeshow, only describe the deviations between observed and expected mortality, without any description of the direction, extent and risk subgroups affected by these deviations. In the same way, they average the risk of patients in each decile and do not use the whole information carried by the individual patient [28,29].

## Conclusions

This study highlights the importance of external validation of prognostic scores in intermediate care, before the routine application of new severity indexes.

SAPS II and SAPS 3 showed similar calibration performance in ImCU, but SAPS II showed better discrimination in terms of SMR. 
